# Supplementary material for: Three learning stages and accuracy–efficiency tradeoff of restricted Boltzmann machines
Source: Nat Commun. 2022 Sep 17;13:5474. doi: 10.1038/s41467-022-33126-x (PMC9482660; doi:10.1038/s41467-022-33126-x)
Supplement: Supplementary file 1 — Supplementary Information [file 41467_2022_33126_MOESM1_ESM.pdf]

Supplementary Information for "Three Learning Stages and Accuracy-Efficiency Tradeoff of Restricted Boltzmann Machines"

Lennart Dabelow[1] and Masahito Ueda[2, 1]

[1]*RIKEN Center for Emergent Matter Science (CEMS), Wako, Saitama 351-0198, Japan*
[2]*Department of Physics and Institute for Physics of Intelligence, Graduate School of Science, The University of Tokyo, Bunkyo-ku, Tokyo 113-0033, Japan*

Labels of equations, figures, tables, and references in these Supplementary Notes are prefixed by a capital letter "S" (e.g., Fig. S1, Eq. (S3)). Any plain labels (e.g., Fig. 1, Eq. (3)) refer to the corresponding items in the main text.

S1. TRAINING DETAILS

With the exception of Fig. 3e, the data presented in the main text were obtained from RBMs trained with a stochastic gradient descent scheme based on the ideal gradient descent updates from Eq. (4) of the main text and utilizing contrastive divergence (CD, see Refs. [S1, S2]) or persistent contrastive divergence (PCD, see Ref. [S3]) to approximate the model averages. Concretely, the training dataset $S = \{\tilde{x}^{(1)}, \ldots, \tilde{x}^{(|S|)}\}$ was partitioned randomly into $s := \frac{|S|}{B}$ minibatches $S_1, \ldots, S_s$ of size B at the beginning of each epoch t. We recall that Markov chains of the form

$$x^{(0)} \to h^{(0)} \to x^{(1)} \to h^{(1)} \to \cdots \tag{S1}$$

are employed to assess the model distribution approximately (cf. Eq. (5) of the main text). We denote a particular (random) realization of $x^{(n)}$ and $h^{(n)}$ for a chain initiated at a (fixed) $x^{(0)} = \tilde{x}$ by $\hat{x}^{(n)}(\tilde{x})$ and $\hat{h}^{(n)}(\tilde{x})$, respectively. In CD, the updates (4) are then approximated as

$$\begin{aligned} &w_{ij}(t + \tfrac{r}{s}) - w_{ij}(t + \tfrac{r-1}{s}) \\ &\quad = \frac{\eta}{B} \sum_{\tilde{x} \in S_r} \left[\tilde{x}_i \hat{h}_j^{(0)}(\tilde{x}) - \hat{x}_i^{(n_{\mathrm{CD}})}(\tilde{x}) \hat{h}_j^{(n_{\mathrm{CD}})}(\tilde{x}) \right], \end{aligned} \tag{S2a}$$

$$\begin{aligned} &a_i(t + \tfrac{r}{s}) - a_i(t + \tfrac{r-1}{s}) \\ &\quad = \frac{\eta}{B} \sum_{\tilde{x} \in S_r} \left[\tilde{x}_i - \hat{x}_i^{(n_{\mathrm{CD}})}(\tilde{x}) \right], \end{aligned} \tag{S2b}$$

$$\begin{aligned} &b_j(t + \tfrac{r}{s}) - b_j(t + \tfrac{r-1}{s}) \\ &\quad = \frac{\eta}{B} \sum_{\tilde{x} \in S_r} \left[\hat{h}_j^{(0)}(\tilde{x}) - \hat{h}_j^{(n_{\mathrm{CD}})}(\tilde{x}) \right] \end{aligned} \tag{S2c}$$

for $r = 1, \ldots, s$. In essence, the model averages in (4) are thus approximated by empirical averages over samples from Markov chains (S1) that are initialized with a training sample $x^{(0)} = \tilde{x} \in S$. If Δ_θ is sufficiently small, such $x^{(0)}$ from S may already be a reasonable approximation for a sample from $\hat{p}_\theta(x)$, and the chain generates a new (but correlated) sample. In the beginning of training, when $\hat{p}_\theta(x)$ is still far from $p(x)$, such an initialization of the chains is less justified, but it is found to work in practice [S2, S4, S5], not least because the mixing and autocorrelation times of the chains are typically small as well in this case.

In PCD, the updates (4) are approximated as

$$\begin{aligned} &w_{ij}(t + \tfrac{r}{s}) - w_{ij}(t + \tfrac{r-1}{s}) \\ &\quad = \eta \left[\frac{1}{B} \sum_{\tilde{x} \in S_r} \tilde{x}_i \hat{h}_j^{(0)}(\tilde{x}) - \frac{1}{L} \sum_{x' \in Q(t + \frac{r}{s})} x'_i \hat{h}_j^{(0)}(x') \right], \end{aligned} \tag{S3a}$$

$$\begin{aligned} &a_i(t + \tfrac{r}{s}) - a_i(t + \tfrac{r-1}{s}) \\ &\quad = \eta \left[\frac{1}{B} \sum_{\tilde{x} \in S_r} \tilde{x}_i - \frac{1}{L} \sum_{x' \in Q(t + \frac{r}{s})} x'_i \right], \end{aligned} \tag{S3b}$$

$$\begin{aligned} &b_j(t + \tfrac{r}{s}) - b_j(t + \tfrac{r-1}{s}) \\ &\quad = \eta \left[\frac{1}{B} \sum_{\tilde{x} \in S_r} \hat{h}_j^{(0)}(\tilde{x}) - \frac{1}{L} \sum_{x' \in Q(t + \frac{r}{s})} \hat{h}_j^{(0)}(x') \right], \end{aligned} \tag{S3c}$$

$$Q(t + \tfrac{r}{s}) = \left\{ \hat{x}^{(n_{\mathrm{CD}})}(x') : x' \in Q(t + \tfrac{r-1}{s}) \right\} \tag{S3d}$$

for $r = 1, \ldots, s$, where $Q(0)$ is a set of random, independent configurations of the visible units and $L := |Q(0)|$. We always use $L = B$. Hence the model averages are approximated by empirical averages over samples from Markov chains (S1) that are initialized with (or, in other words, continued from) samples of the previous update step, i.e., the chains are *persistent*. Note, however, that the model distribution used to generate (or advance) the chains changes from step to step as the model parameters θ change. If these parameter updates are sufficiently small and the initial configurations $x' \in Q(0)$ emulate samples from the initial model $\hat{p}_{\theta(0)}(x)$, the chains approximately reflect the gradually evolving stationary model distribution throughout the training process. However, subsequent samples are generally not independent, especially if $n_{\mathrm{CD}} \lesssim \tau_\theta$.

The total number of training epochs in Figs. 2–4 of the main paper as well as in this Supplementary Information varies between 2×10^4 and 3×10^6, depending on the time needed until improvement of the accuracy could no longer be observed and extending reasonably far beyond it to capture the degradation regime. As mentioned in the main text, the machines were initialized

by drawing the parameters $\theta_k(0)$ independently from a normal distribution $\mathcal{N}(\mu, \sigma)$ of mean μ and standard deviation σ, namely $w_{ij}(0) \sim \mathcal{N}(0, 10^{-2})$ and $a_i(0), b_j(0) \sim \mathcal{N}(0, 10^{-1})$ (Figs. 2, 3, and 4b,c) or $a_i = b_j = 0$ (Fig. 4e). Figs. 2 and 3 show averages over 5 independent repetitions of the experiment for each hyperparameter configuration. Fig. 4 shows results for single machines.

For the data in Fig. 3e, we utilized—as described in the main text—the full target distribution $p(x)$ and the exact n-step CD model distribution $\hat{p}_\theta^{(n)}$ or the full model distribution $\hat{p}_\theta(x)$ for the expectation values. Moreover, we employed the continuous-time limit of the update equations (4). Hence the evolution of the weights is governed by the differential equations

$$\dot{w}_{ij}(t) = \sum_{x,h} x_i h_j \, \hat{p}_{\theta(t)}(h \,|\, x) \left[p(x) - \hat{p}_{\theta(t)}^{(n)}(x) \right], \tag{S4a}$$

$$\dot{a}_i(t) = \sum_{x} x_i \left[p(x) - \hat{p}_{\theta(t)}^{(n)}(x) \right], \tag{S4b}$$

$$\dot{b}_j(t) = \sum_{x,h} h_j \, \hat{p}_{\theta(t)}(h \,|\, x) \left[p(x) - \hat{p}_{\theta(t)}^{(n)}(x) \right], \tag{S4c}$$

where the dots indicate derivatives with respect to t and

$$\hat{p}_\theta^{(n)}(x) := \sum_{x',h'} \hat{p}_\theta(x \,|\, h') \, \hat{p}_\theta(h' \,|\, x') \hat{p}_\theta^{(n-1)}(x'), \tag{S5}$$

$$\hat{p}_\theta^{(0)}(x) := p(x), \tag{S6}$$

and $\hat{p}_\theta^{(\infty)}(x) \equiv \hat{p}_\theta(x)$. We then integrated Eqs. (S4) numerically (starting from random initial conditions as before) using *Mathematica*'s `NDSolve` routine.

S2. EXAMPLE TASKS

A. Transverse-field Ising chain

The transverse-field Ising chain (TFIC) with M sites is defined by the Hamiltonian

$$H = -\frac{1}{2} \sum_{i=0}^{M-1} \left(\sigma_i^x \sigma_{i+1}^x + g\, \sigma_i^z \right) \tag{S7}$$

with periodic boundary conditions, $\sigma_{i+M}^\gamma = \sigma_i^\gamma$, where σ_i^γ ($\gamma = x, y, z$) are the Pauli matrices acting on site i. The corresponding spin raising and lowering operators are $\sigma_i^\pm := \frac{1}{2}(\sigma_i^x \pm \mathrm{i}\sigma_i^y)$.

The Hamiltonian can be diagonalized by the following sequence of transformations: the Jordan-Wigner transformation

$$c_i := P_i \sigma_i^- \tag{S8}$$

with $P_i := \prod_{j=0}^{i-1} (-\sigma_i^z)$, the Fourier transformation

$$\tilde{c}_k := \frac{1}{\sqrt{L}} \sum_i \mathrm{e}^{-\mathrm{i}ki} c_i \tag{S9}$$

TABLE S1. Entropy $S(p)$ and total correlation $C_{\mathrm{tot}}(p)$ of the TFIC ground-state distribution in the σ^z and σ^x bases for various values of the magnetic-field strength g.

	σ^z basis		σ^x basis	
g	$S(p)$	$C_{\mathrm{tot}}(p)$	$S(p)$	$C_{\mathrm{tot}}(p)$
0.5	12.48	0.705	1.216	12.65
0.8	10.99	0.824	5.062	8.801
1	8.028	1.441	8.721	5.142
1.2	4.808	1.891	11.38	2.478
2	1.760	1.134	13.17	0.689
4	0.523	0.400	13.70	0.160

with fermionic (bosonic) Matsubara frequencies k if the total particle number $\sum_i c_i^\dagger c_i$ is even (odd), and the Bogoliubov transformation

$$\eta_k := u_k \tilde{c}_k - v_k \tilde{c}_{-k}^\dagger \tag{S10}$$

with $u_k := (\varepsilon_k + \alpha_k)/\omega_k$, $v_k := \mathrm{i}\beta_k/\omega_k$, $\alpha_k := -2J(g + \cos k)$, $\beta_k := 2J \sin k$, $\varepsilon_k^2 := \alpha_k^2 + \beta_k^2$, $\omega_k^2 := 2\varepsilon_k(\varepsilon_k + \alpha_k)$; see, for example, Ref. [S6]. The resulting Hamiltonian is

$$H = \sum_k \varepsilon_k \left(\eta_k^\dagger \eta_k - \tfrac{1}{2} \right) \tag{S11}$$

in the sector with an even number of particles, to which the ground state belongs. This ground state can be constructed as

$$|\psi\rangle := \prod_k \frac{1}{|v_k|} \eta_k \eta_{-k} |{\downarrow} \cdots {\downarrow}\rangle \tag{S12}$$

from the state $|{\downarrow} \cdots {\downarrow}\rangle$ with all spins down in the σ^z basis [S6].

Moreover, by adjusting the global phase, it can be written such that $\psi(x) := \langle x_0 \cdots x_{M-1} | \psi \rangle$ is real-valued and nonnegative when $|x_0 \cdots x_{M-1}\rangle$ is a basis state in the σ^z or σ^x bases. For our quantum-state tomography task of the ground-state wave function from measurements in either of the two bases, we can therefore take the target distribution as $p(x) := \psi(x)^2$ and do not need additional modifications of the RBM model to facilitate the phase reconstruction [S7].

Tab. S1 lists the entropy $S(p) := -\sum_x p(x) \ln p(x)$ and total correlation $C_{\mathrm{tot}}(p)$ (cf. Eq. (8) of the main text) of the target distribution obtained for various values of g.

B. Hook-pattern images

We consider $L \times L$ images $x = (x_{i_1,i_2})_{i_1,i_2=0}^{L-1}$ whose pixels $x_i = x_{i_1,i_2}$ are either black ($x_i = 0$) or white ($x_i = 1$). We denote the set of all $2^{L \times L}$ of these images by $\mathcal{K}$.

The characteristic feature of the target distribution $p(x)$ is a "hook" pattern comprised of a total of 15 pixels, cf. Fig. 3a. We assume periodic boundary conditions

$(x_{i,j} = x_{i+L,j} = x_{i,j+L})$ and denote the max-distance on the image grid by

$$d_\infty(i,j) = \max_{\mu=1,2} \min\{|i_\mu - j_\mu|, L - |i_\mu - j_\mu|\}\,. \quad \text{(S13)}$$

Similarly, for a set of pixel positions (sites) I and a single site j, define $d_\infty(I,j) := \min\{d_\infty(i,j) : i \in I\}$. An image x then shows the "hook" pattern at site i if the pixels at sites $I_0(i) := \{(i_1,i_2),(i_1-1,i_2),(i_1,i_2+1)\}$ are white *and* the pixels at sites $I_1(i) := \{j : d_\infty(I_0(i),j) = 1\}$ are black. In other words, $x_j = 1$ for all $j \in I_0(i)$ and $x_j = 0$ for all $j \in I_1(i)$. The remaining $L^2 - 15$ pixels at sites $I_{\geq 2}(i) := \{j : d_\infty(I_0(i),j) \geq 2\}$ can take arbitrary values. We denote the set of all images with a "hook" pattern at an arbitrary site by $\mathcal{K}_\mathrm{H}$. In the main text, we choose $L = 5$; examples are shown in Fig. 3a. Note that there are a total of $|\mathcal{K}_\mathrm{H}| = L^2 \times 2^{L^2-15} = 25\,600$ images exhibiting the pattern, which is a fraction of $|\mathcal{K}_\mathrm{H}|/|\mathcal{K}| = 25 \times 2^{-15} \approx 0.08\,\%$ of all 5×5 images.

The target distribution picks a random location $i = (i_1,i_2)$ for the hook pattern and activates all remaining pixels in $I_{\geq 2}(i)$ with probability $q = \frac{1}{10}$. Hence

$$p(x) = \mathbb{1}_{\mathcal{K}_\mathrm{H}}(x) \sum_i \prod_{j \in I_{\geq 2}(i)} [x_j\, q + (1-x_j)(1-q)]\,, \quad \text{(S14)}$$

where $\mathbb{1}_S$ is the indicator function of the set S, i.e., $\mathbb{1}_S(x) = 1$ if $x \in S$ and 0 otherwise.

The entropy is $S(p) \approx 6.47$ and the total correlation is $C_\mathrm{tot}(p) \approx 4.53$.

The one-dimensional, smaller distributions from Fig. 3d are structurally similar, but involve a core pattern of white pixels at sites $I_0(i)$ of only one $(M = 4)$ or two $(M = 5)$ pixels, surrounded by one-site boundaries $I_1(i)$ of black pixels in either direction, with the remaining pixel in $I_{\geq 2}(i)$ being arbitrary again.

C. Digit images

We consider $H \times W$ images $x = (x_{i_1,i_2})$ with pixel values $x_i = x_{i_1,i_2} \in \{0,1\}$ and denote the set of all such images by $\mathcal{K}$.

The target distribution involves images showing one of ten patterns representing the digits 0 through 9 (cf. Fig. 4a of the main text). The digit patterns consist of a core block of fixed black or white pixels (black or white in Fig. 4a) as well as a boundary (gray-shaded in Fig. 4a) which may either be represented by black pixels or by the image frame (i.e., the gray pixels may lie "out of bounds"). No periodic boundary conditions are assumed. The remaining pixels outside of the respective pattern may take arbitrary values. Due to the different sizes of the digit patterns, the number of possible images for each pattern is different in general. We denote the set of all images with a "k" pattern by $\mathcal{K}_k$. For $H = 7$ and $W = 5$, which is our choice for the data shown in Fig. 4a–c of the main text, the resulting cardinalities of the sets $\mathcal{K}_k$ are summarized in Tab. S2.

TABLE S2. Cardinalities of the sets $\mathcal{K}_k$ of digit images. In total, $|\mathcal{K}_\mathrm{PN}| = 40\,507\,353$ out of the total of $2^{7\times 5} = 34\,359\,738\,368$ possible images show digit patterns.

k	$\lvert\mathcal{K}_k\rvert$	k	$\lvert\mathcal{K}_k\rvert$
0	8 513	5	565 391
1	38 558 138	6	56 464
2	565 391	7	66 624
3	565 391	8	8 513
4	56 464	9	56 464

The target distribution selects a pattern k uniformly at random, $p(x \in \mathcal{K}_k) = \frac{1}{10}\mathbb{1}_{\bigcup_l \mathcal{K}_l}(x)$. The selected pattern is then placed at a random position $i = (i_1,i_2)$ with uniform probability $p(x \in \mathcal{K}_{k,i} \,|\, x \in \mathcal{K}_k)$, where $\mathcal{K}_{k,i}$ is the set of all images showing pattern k at position i. Due to the different pattern shapes, again, the admissible positions are generally different for different patterns. Denoting the set of all pixel sites that are not part of the pattern k at position i by $\bar{I}(k,i)$, the remaining pixels represent noise and are white independently with probability $q = \frac{1}{10}$, i.e., $p(x_j = 1 | j \in \bar{I}(i,k)) = q$. The total probability of a given image x is thus

$$p(x) = \sum_k p(x \in \mathcal{K}_k) \sum_i p(x \in \mathcal{K}_{k,i} \,|\, x \in \mathcal{K}_k) \times \prod_{j \in I(k,i)} [x_j\, q + (1-x_j)(1-q)]\,. \quad \text{(S15)}$$

Note that an image can "accidentally" have patterns at multiple positions; for the image sizes we adopted, in particular, this can happen if one of them is the "1" pattern.

The entropy of this distribution is $S(p) \approx 8.35$ and the total correlation is $C_\mathrm{tot}(p) \approx 11.67$.

For the empirical loss measure $\tilde{\Delta}_\sigma^{(T,\hat{T})}$ in Fig. 4c, the parameter σ is determined by minimizing $\tilde{\Delta}_\sigma^{(T,S)}$ between the test and training datasets of $|T| = 10\,000$ and $|S| = 50\,000$ samples, respectively. From the relationship shown in Fig. S1a, we find $\tilde{\Delta}_\sigma^{(T,S)} \approx 1.354$ at $\sigma \approx 0.32$ for the minimum.

D. MNIST

We use the MNIST dataset of 28×28-pixel images of handwritten digits with its standard splitting into $|S| = 60\,000$ training and $|T| = 10\,000$ test images [S8]. Each subset contains equal fractions of representations for each digit. The grayscale pixel values $z_i \in \{0,1,\ldots,255\}$ are preprocessed as $x_i = \lfloor \frac{z_i}{128} \rfloor$ to obtain a binary dataset. The entropies of the thus-obtained training and test datasets are $S(\tilde{p}(\,\cdot\,;S)) \approx 11.00$ and $S(\tilde{p}(\,\cdot\,;T)) \approx 9.21$, and their total correlations are $C_\mathrm{tot}(\tilde{p}(\,\cdot\,;S)) \approx 195.0$ and $C_\mathrm{tot}(\tilde{p}(\,\cdot\,;T)) \approx 196.5$, respectively.

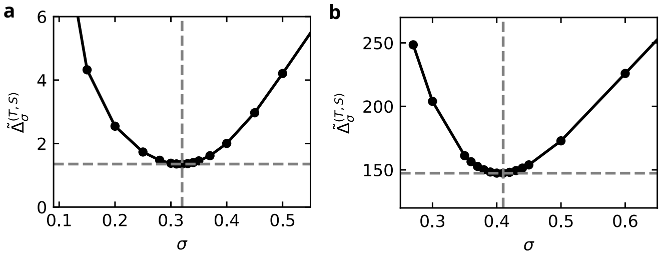

FIG. S1. Kullback-Leibler divergence $\tilde{\Delta}_\sigma^{(T,S)} := D_{\mathrm{KL}}(\tilde{p}(\,\cdot\,;T)||\tilde{p}_\sigma(\,\cdot\,;S))$ between the empirical distribution of the test data $\tilde{p}(x;T)$ and the Gaussian-smoothened empirical distribution $\tilde{p}_\sigma(x;S)$ of the training data as a function of the smoothening width parameter σ. The value of σ for which $\tilde{\Delta}_\sigma^{(T,S)}$ becomes minimal is adopted in Fig. 4 to calculate the empirical loss measure $\tilde{\Delta}_\sigma^{(T,\hat{T})}$.

Similarly as before, the parameter σ for $\tilde{\Delta}_\sigma^{(T,\hat{T})}$ in Fig. 4e is chosen such that $\tilde{\Delta}_\sigma^{(T,S)}$ becomes minimal. As shown in Fig. S1b, we obtain $\tilde{\Delta}_\sigma^{(T,S)} \approx 147.4$ at $\sigma \approx 0.41$ for that minimum.

S3. INITIALIZATION SCHEMES

As argued in the main text, the most natural way to initialize the RBM parameters $(\theta_k) = (w_{ij}, a_i, b_j)$ is to assign small random values to them if no information about the target distribution $p(x)$ is available. In the main text, we thus sample the initial θ_k from independent normal distributions $\mathcal{N}(\mu, \sigma)$ with vanishing mean $\mu = 0$ and small (or vanishing) standard deviation σ. In Figs. S2 and S3, we compare the resulting relationship between Δ_θ and τ_θ for other initialization schemes.

One piece of information about the target distribution that is easily accessible is the approximate value of the marginal probabilities $p_i(x_i)$ of the visible units. Hence Hinton [S2] suggests to initialize the visible-unit biases as $a_i = \ln[\nu_i/(1-\nu_i)]$, where $\nu_i := \frac{1}{|S|}\sum_{\tilde{x}\in S}\tilde{x}_i$ is the frequency of $x_i = 1$ in the training dataset S, i.e., $p_i(x_i = 1) \approx \nu_i$. In the numerical examples from the second columns of Figs. S2 and S3, we cap the so-obtained a_i at 2 in absolute value. For the weights and hidden-unit biases, Ref. [S2] suggests using $w_{ij} \sim \mathcal{N}(0, 0.01)$ and $b_j = 0$. Following this scheme, one can shorten the independent-learning period as can be seen in the second columns of Figs. S2 and S3. Nevertheless, we observe the same learning characteristics as for the fully random initialization in the correlation-learning and degradation regimes.

By accident or deliberation, the initial model distribution may already exhibit noticeable but spurious correlations as well. This is examplified in the last three columns of Figs. S2 and S3. Such initial correlations may arise, for instance, if the weights are chosen too large (third columns) or if parameters from a pre-trained machine using a different target distribution are adopted (fourth and fifth columns). In the case of such spurious initial correlations, the machine typically starts further away from the lower bound (7). As training progresses, however, the bound is approached by decreasing both Δ_θ and τ_θ first and eventually showing similar tradeoff characteristics as in the "independent" initialization schemes (first two columns).

S4. AUTOCORRELATION TIMES OF DIFFERENT OBSERVABLES

As explained in the main text (see Methods), different observables $f(x)$ generally exhibit different integrated autocorrelation times $\tau_\theta^{(f)}$. We recall that the latter are defined as

$$\tau_\theta^{(f)} := 1 + 2\sum_{n=1}^{\infty} \frac{g_\theta^{(f)}(n)}{g_\theta^{(f)}(0)}\,, \tag{S16}$$

where

$$g_\theta^{(f)}(n) := \langle f(x^{(0)}) f(x^{(n)})\rangle - \langle f(x^{(0)})\rangle^2 \tag{S17}$$

is the correlation function of $f(x)$ for the Markov chain (S1) initialized in the stationary state $x^{(0)} \sim \hat{p}_\theta(x)$ (cf. Eqs. (15) and (16) in the main text).

The quantity τ_θ from Eq. (6), which is our principal measure of sampling efficiency in the main text, is a weighted average of the autocorrelation times $\tau_\theta^{(x_i)}$ of the individual visible units $f(x) = x_i$ (see Eq. (18)). In the following, we verify that the autocorrelation times $\tau_\theta^{(f)}$ typically scale similarly to τ_θ. More precisely, they are found to be largely proportional to each other. For the power-law bound (7) which quantifies the accuracy–efficiency tradeoff, this changes the constant c on the right-hand side to an observable-dependent $c^{(f)}$. Those constants can no longer be identified with the total correlation $C_{\mathrm{tot}}(p)$ of the full target distribution $p(x)$. Instead, the pertinent reference should be a characteristic of the distribution $p(f(x))$ of the transformed variables, for which, however, it may not always be possible or reasonable to define a "total correlation."

In Fig. S4, we adopt the same setup and RBMs as in the third column of Fig. 2b of the main text (TFIC, σ^z basis, $n_{\mathrm{CD}} = 1$, $|S| = 25\,000$). We plot the integrated autocorrelation times $\tau_\theta^{(f)}$ for various observables against τ_θ. Concretely, the investigated observables are

- the individual visible units,

$$f(x) = x_i\,; \tag{S18a}$$

- the nearest-neighbor correlation function,

$$f(x) = x_i x_{i+1}\,; \tag{S18b}$$

- the next-nearest neighbor correlation function,

$$f(x) = x_i x_{i+2}\,; \tag{S18c}$$

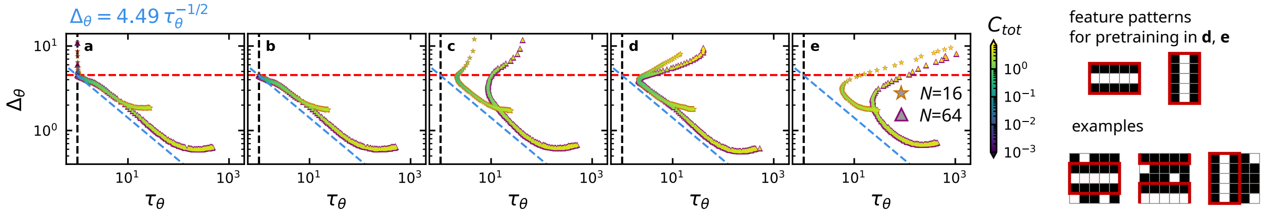

FIG. S2. Exact loss Δ_θ vs. autocorrelation time τ_θ for various initialization schemes of the weights in the pattern recognition task from Fig. 3 (see also Sec. S2 B). Initialization schemes: (a) $w_{ij} \sim \mathcal{N}(0, 0.01)$, $a_i, b_j \sim \mathcal{N}(0, 0.1)$ (similar to the main text); (b) $w_{ij} \sim \mathcal{N}(0, 0.01)$, $a_i = \ln[\nu_i/(1 - \nu_i)]$ with $\nu_i := \frac{1}{|S|}\sum_{\tilde{x}\in S} \tilde{x}_i$ the activation frequency of the ith visible unit in the training dataset S, $b_j = 0$ [S2]; (c) $w_{ij}, a_i, b_j \sim \mathcal{N}(0, 1)$; (d) snapshot after $t = 500$ training epochs of a machine initialized like in (a) and subsequently trained on images with horizontal or vertical line patterns (see sketch) and otherwise identical hyperparameters; (e) similar to (d), but using snapshots after $t = 50\,000$ training epochs. Data points show averages over 5 independent runs for each scheme. Fill colors indicate the total correlation $C_{\mathrm{tot}}(\hat{p}_\theta)$ of the model distribution (see colorbar), border colors and marker types indicate the number of hidden units N (see legend in (e)). Further hyperparameters: CD training with $n_{\mathrm{CD}} = 1$, $\eta = 0.005$, $B = 100$, $|S| = 5000$.

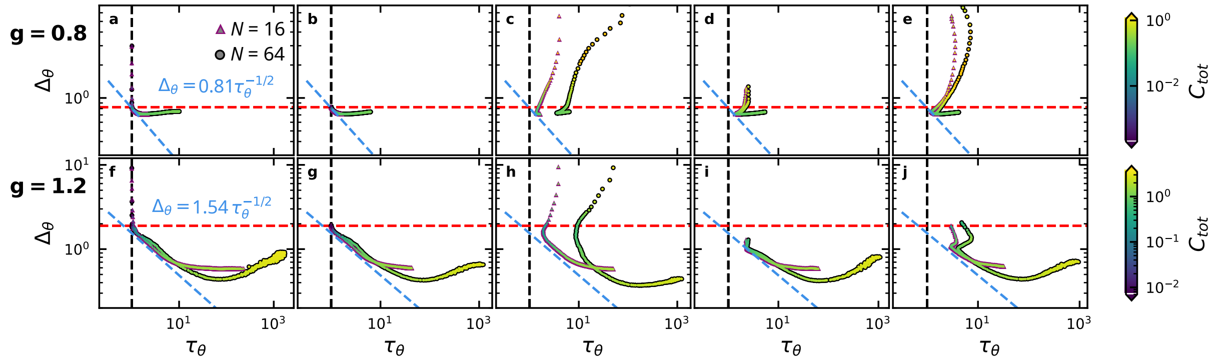

FIG. S3. Exact loss Δ_θ vs. autocorrelation time τ_θ for various initialization schemes of the weights in the ground-state tomography task for the TFIC with $g = 0.8$ (top row) and $g = 1.2$ (bottom row), cf. Fig. 2 and Sec. S2 A). Initialization schemes: (a,f) $w_{ij} \sim \mathcal{N}(0, 0.01)$, $a_i, b_j \sim \mathcal{N}(0, 0.1)$ (similar to the main text); (b,g) $w_{ij} \sim \mathcal{N}(0, 0.01)$, $a_i = \ln[\nu_i/(1 - \nu_i)]$ with $\nu_i := \frac{1}{|S|}\sum_{\tilde{x}\in S} \tilde{x}_i$ the activation frequency of the ith visible unit in the training dataset S, $b_j = 0$ [S2]; (c,h) $w_{ij}, a_i, b_j \sim \mathcal{N}(0, 1)$; (d,i) snapshot after $t = 5000$ training epochs of a machine initialized like in (a,f) and subsequently trained to learn the ground state for $g = 1$ and otherwise identical hyperparameters; (e,j) similar to (d,i), but using snapshots after $t = 5000$ training epochs when learning the $g = 2$ ground state. Data points show averages over 5 independent runs for each scheme. Fill colors indicate the total correlation $C_{\mathrm{tot}}(\hat{p}_\theta)$ of the model distribution (see colorbars), border colors and marker types indicate the number of hidden units N (see legend in (a)). Further hyperparameters: CD training with $n_{\mathrm{CD}} = 1$, $\eta = 0.001$, $B = 100$, $|S| = 25\,000$.

- the 3-point nearest-neighbor correlation function,

$$f(x) = x_i x_{i+1} x_{i+2}\,. \tag{S18d}$$

Note that, due to translational invariance of the target distribution $p(x)$, all those observables should be independent of the reference index $i \in \{1, \ldots, M\}$. However, the learned model distribution $\hat{p}_\theta(x)$ may not fully reflect this symmetry. The autocorrelation time $\tau_\theta^{(f)}$ shown in Fig. S4 is therefore averaged over all indices i. We also show the autocorrelation time for the general 2-point correlation function

$$f(x) = x_{i_1} x_{i_2}\,, \quad i_1, i_2 \in \{1, \ldots M\}\,, \tag{S19}$$

again averaged over all pairs (i_1, i_2). (Note that this observable still depends on the difference $i_1 - i_2$ though.) Finally, we also include autocorrelation times for the mean over all visible units,

$$f(x) = \frac{1}{M}\sum_i x_i\,. \tag{S20}$$

We point out that there are RBM configurations for which we did not obtain a reliable estimate of τ_θ or $\tau_\theta^{(f)}$ within the maximally admitted number of sampling steps (see also Methods in the main text). Therefore, the data points are sparser for $N = 64$, in particular.

As mentioned above, the results indicate that $\tau_\theta^{(f)}$ is usually proportional to $f(x)$. With regard to the seemingly largest (relative) deviations in the top-left panel, we observe that the autocorrelation times are generally very small in this case. We also remark that, due to translation invariance, the trained models should satisfy $\tau_\theta^{(x_i)} = \tau_\theta$ if they realized this symmetry exactly. Devia-

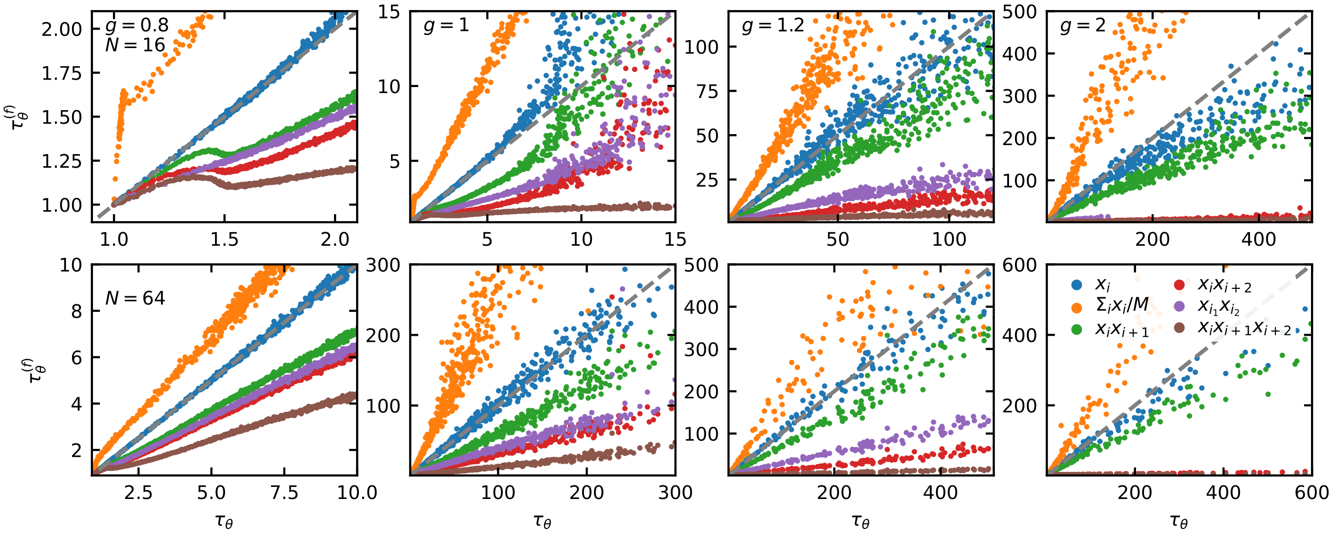

FIG. S4. Integrated autocorrelation times $\tau_\theta^{(f)}$ (cf. Eqs. (S18)–(S20)) for various observables $f(x)$ (see legend in the bottom-right panel) vs. sampling efficiency τ_θ in the transverse-field Ising chain (TFIC, cf. Fig. 2 and Sec. S2 A) in the σ^z basis. The panels show results for different external fields g (cf. Eq. (S7)) in the columns and different numbers of hidden units N in the rows as indicated. Hyperparameters: $n_{\mathrm{CD}} = 1$, $\eta = 10^{-3}$, $B = 100$, $|S| = 25\,000$. For observables depending on the visible-unit indices i, i_1, i_2, the data are averaged over all those indices. Furthermore, as in the main text, each data point is an average over five independently trained RBMs at a fixed training epoch t. The gray dashed line shows the function $\tau_\theta^{(f)} = \tau_\theta$ for reference.

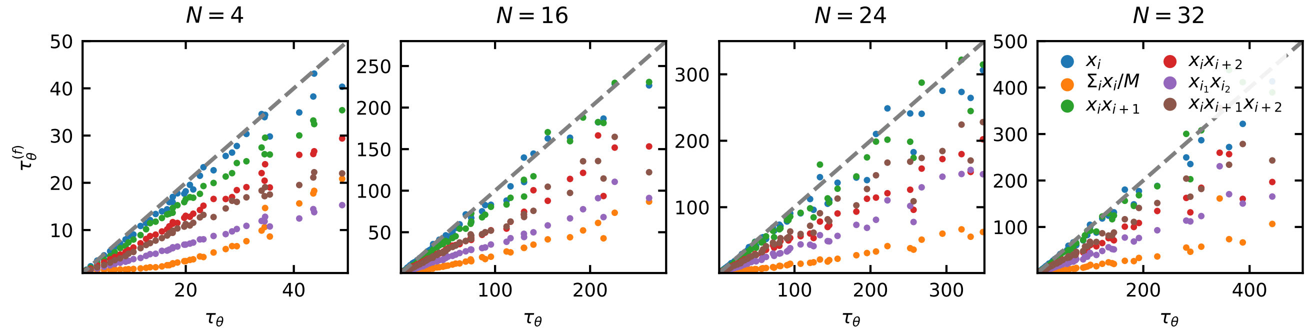

FIG. S5. Integrated autocorrelation times $\tau_\theta^{(f)}$ (cf. Eqs. (S18)–(S20)) for various observables $f(x)$ (see legend in the right-most panel) vs. sampling efficiency τ_θ in the digit-pattern example from Fig. 4a–c and Sec. S2 C. The panels show results for different numbers of hidden units N as indicated. Hyperparameters: $n_{\mathrm{CD}} = 1$, $\eta = 0.005$, $B = 100$, $|S| = 50\,000$. For observables depending on the visible-unit indices i, i_1, i_2, the data are averaged over all those indices. Furthermore, as in the main text, each data point is an average over five independently trained RBMs at a fixed training epoch t. The gray dashed line shows the function $\tau_\theta^{(f)} = \tau_\theta$ for reference.

tions from this ideal behavior can hint at overfitting and insufficient expressivity.

The same conclusions can be drawn from Fig. S5, which shows autocorrelation times for different observables in the digit-pattern example from Fig. 4a–c and Sec. S2 C. We remark that, due to the distinct geometry, some of the observables do not have the same physical meaning as in the TFIC example; for instance, $x_i x_{i+1}$ is a correlation function between nearest neighbors along the columns only.

Finally, for a more direct visualization of correlations between samples of Markov chains like (S1), we show snapshots from such chains for RBMs trained on the MNIST dataset in Fig. S6. The autocorrelation-time estimate τ_θ conforms nicely with the number of steps needed to reach a sample that looks "new" or "uncorrelated" to the naked eye. The adequacy of τ_θ to quantify correlations between Markov-chain samples and to estimate the additional steps required to obtain an independent sample is thus reinforced.

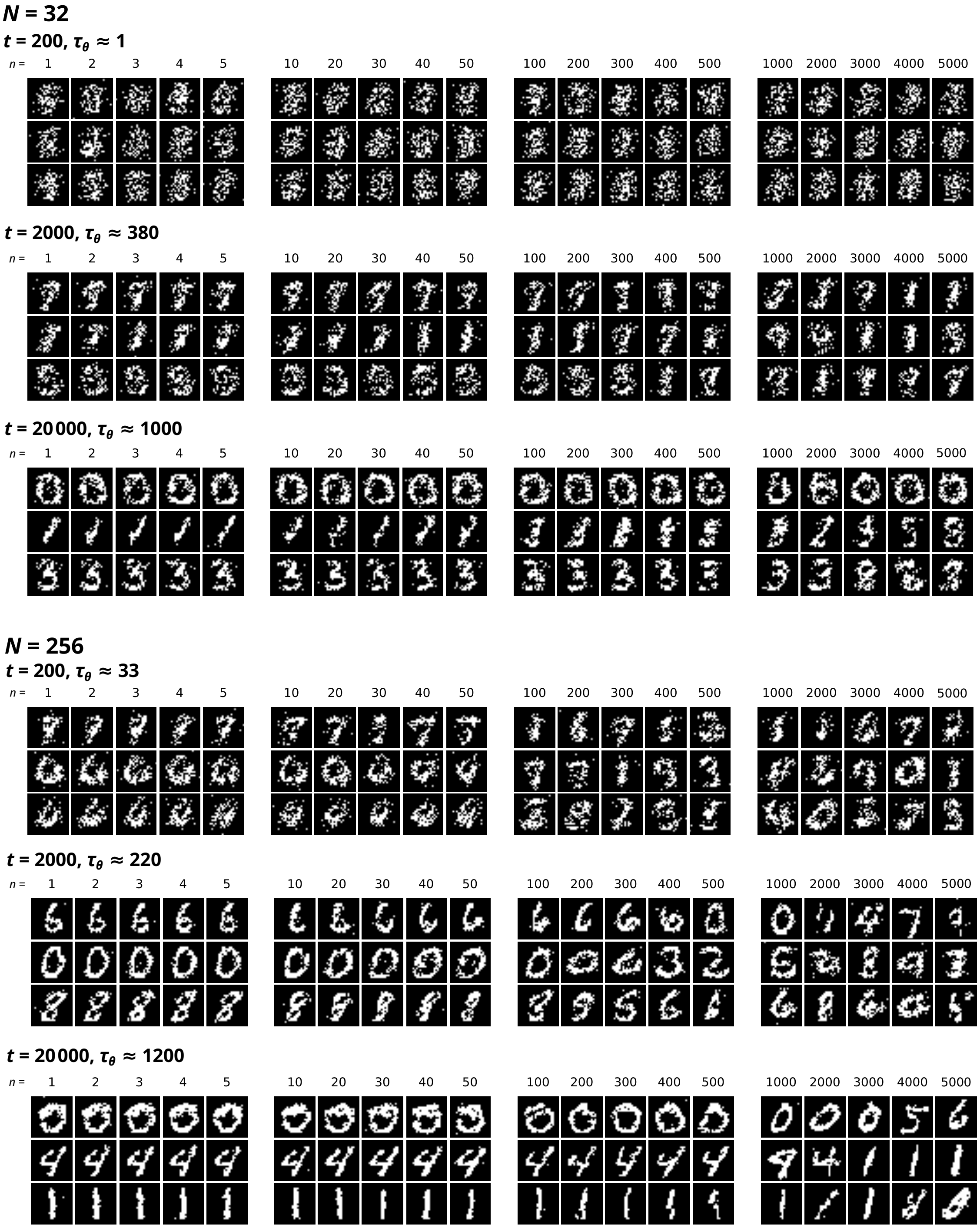

FIG. S6. Example Markov chains obtained from RBMs trained on the MNIST dataset with the PCD algorithm and $n_{\mathrm{CD}} = 1$, $\eta = 10^{-4}$, $B = 100$ for different numbers of hidden units N and training epochs t. All chains were initialized with a uniform random distribution of the visible units x_i [i.e., $p(x_i = 0) = p(x_i = 1) = \frac{1}{2}$] and subsequently thermalized for 2×10^6 steps before recording starts.

S5. EXTENDED MECHANISM

We expand on aspects of the discussion in the section "Mechanism" from the main text.

A. Minimal loss for independent units

As stated around Eq. (8) of the main text, the exact loss $\Delta_\theta = D_{\mathrm{KL}}(p||\hat{p}_\theta)$ is bounded from below by the total correlation $C_{\mathrm{tot}}(p)$ if $\hat{p}_\theta$ consists of independent units.

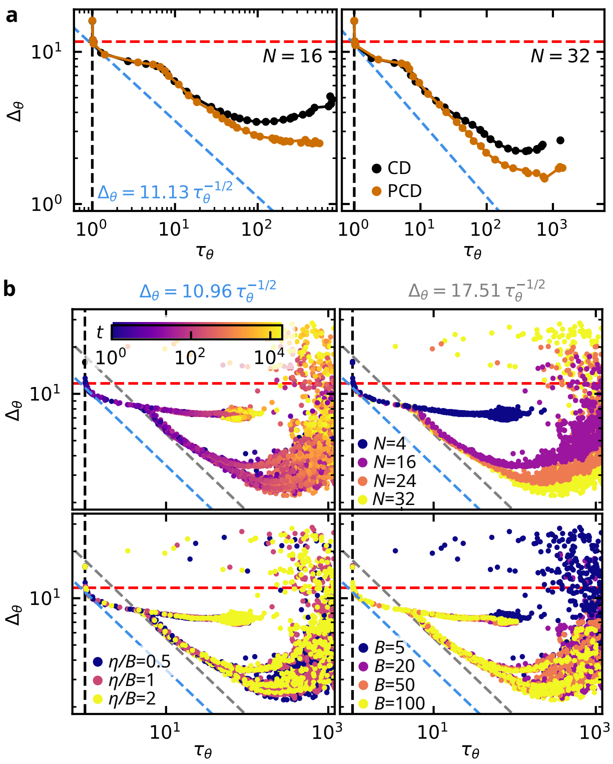

FIG. S7. Exact loss Δ_θ vs. autocorrelation time τ_θ for RBMs in the digit-generator example from Fig. 4a–c and Sec. S2 C. Training used a dataset of $|S| = 50\,000$ samples. Data points are averages over 5 independent runs. (a) Comparison of ordinary contrastive divergence (CD) and persistent contrastive divergence (PCD) with $n_{\mathrm{CD}} = 1$, $\eta = 0.005$, $B = 100$ for $N = 16$ (left) and $N = 32$ (right). (b) CD training with $n_{\mathrm{CD}} = 1$ and $N = 4, 16, 24, 32$, $\eta/B = 0.5, 1, 2$, $B = 5, 20, 50, 100$. All panels show the same data points, but highlight different hyperparameter dependencies by color and brightness as indicated. Overlapping curves of different colors thus signal that the learning characteristics are indepenent of the respective hyperparameter.

Indeed, if $\hat{p}(x) := \prod_i \hat{p}_i(x_i)$, we can make the following decomposition:

$$
\begin{aligned}
D_{\mathrm{KL}}(p||\hat{p}) &= \sum_x p(x) \left[\log \frac{p(x)}{\prod_i p_i(x_i)} + \log \frac{\prod_i p_i(x_i)}{\prod_i \hat{p}_i(x_i)} \right] \\
&= \sum_x p(x) \log \frac{p(x)}{\prod_i p_i(x_i)} \\
&\quad + \sum_i \sum_{x_i} p_i(x_i) \log \frac{p_i(x_i)}{\hat{p}_i(x_i)} \\
&= C_{\mathrm{tot}}(p) + \sum_i D_{\mathrm{KL}}(p_i||\hat{p}_i)\,.
\end{aligned}
$$

Recalling the definition (8) and the fact that $D_{\mathrm{KL}}(p||q) \geq 0$ for arbitrary distributions p and q, we can conclude that $D_{\mathrm{KL}}(p||\hat{p}) \geq C_{\mathrm{tot}}(p)$.

B. Hyperparameter independence of the tradeoff relation

In the main text, we argued that the relationship between Δ_θ and τ_θ in the independent- and correlation-learning regimes is essentially independent of the basic RBM hyperparameters, including the learning rate η, the batch size B, the number of training samples $|S|$, the number of hidden units N, and the approximation scheme for model averages during training (CD vs. PCD and their order n_{CD}). Further evidence for this insensitivity is provided in Fig. S7 for the digit-generator example from Fig. 4a–c and Sec. S2 C.

We emphasize that the choice of appropriate hyperparameters is still important, because it affects the stability of training and the onset of the degradation regime, meaning that poor choices can lead to early deterioration of the RBMs.

C. Scaling of weights and correlations

The magnitude $|\theta_k|$ of the RBM parameters typically grows during training. A distinctive property of many "real-world" machine-learning problems is that the target distribution is sparse, meaning that most states x have vanishing or at least very small probability $p(x)$. For example, the overwhelming majority of all possible images with a given number of pixels will not display "realistic" motifs (e.g., digits, letters, animals, clothes, buildings, ...). This characteristic is at odds with the RBM model family, which assigns a finite probability $\hat{p}_\theta(x) > 0$ to all states x. To suppress the unlikely states, many of the parameters θ_k have to take large absolute values.

As argued around Eq. (11) and illustrated in Fig. S8, this usually increases the correlations between subsequent Markov-chain samples. Notably, correlations between visible and hidden units, and thus between two (or more) visible units, arise only if $|w_{ij}| > 0$. Hence larger $|\theta_k|$ and larger $|w_{ij}|$ in particular hint at larger autocorrelation times τ_θ. Note, however, that $|w_{ij}| > 0$ for some i, j is not sufficient to obtain correlations between different visible units; to this end, two visible units x_{i_1} and x_{i_2} must be coupled to the same hidden unit h_j, i.e., both $|w_{i_1 j}| > 0$ and $|w_{i_2 j}| > 0$ must hold.

Since it is computationally demanding to estimate τ_θ reliably, the standard deviation $\sigma_w := (\frac{1}{MN-1} \sum_{i,j} w_{ij}^2)^{1/2}$ can be considered as a more accessible indicator of growing correlations in practice. In Fig. S8, we show the mutual dependence of τ_θ and σ_w for the examples from Sec. S2 A–S2 C and various numbers of hidden units N. We observe that the two quantities are indeed positively correlated, which confirms, in particular, that τ_θ typically grows with the magnitude of the weights.

The necessity to increase the RBM parameters θ_k in magnitude in order to represent sparse distributions with many "inactive" states x such that $p(x) = 0$ is also

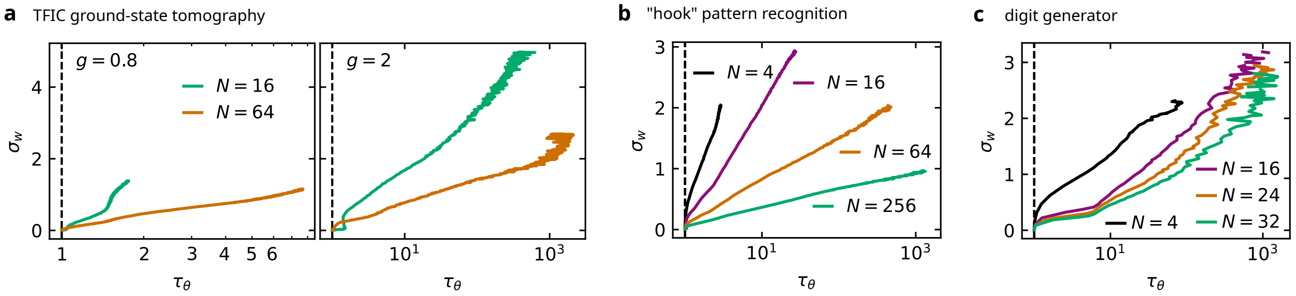

FIG. S8. Standard deviation $\sigma_w := (\frac{1}{MN-1}\sum_{i,j} w_{ij}^2)^{1/2}$ of the weights w_{ij} vs. integrated autocorrelation time τ_θ for RBMs trained with contrastive divergence of order $n_{\mathrm{CD}} = 1$ on three different problems. (a) Ground-state tomography in the transverse-field Ising chain of $M = 20$ sites (cf. Fig. 2 and Sec. S2 A), $|S| = 25\,000$, $\eta = 10^{-3}$, $B = 100$, different N and g as indicated. (b) Pattern-recognition task (cf. Fig. 3 and Sec. S2 B), $|S| = 5000$, $\eta = 0.005$, $B = 100$, different N as indicated. (c) Digit generator (cf. Fig. 4a–c and Sec. S2 C), $|S| = 50\,000$, $\eta = 0.005$, $B = 100$, different N as indicated.

one possible hint at the source of the different α values observed in Fig. 2d compared with Fig. 2b (and also Figs. 3b and 4b,c). The image distributions from Figs. 3a–c and 4b,c all have a large fraction of such "inactive" states (99.92 % and 99.88 %, respectively, see Secs. S2 B and S2 C). Likewise, the TFIC ground state in the σ^z basis (when $\alpha = \frac{1}{2}$) has $p(x) = 0$ for half of the states by symmetry (see Sec. S2 A and S5 D), whereas no such inactive states exist in the σ^x representation (when $\alpha = 6 \ldots 8$).

Within the examples from Fig. 2d, the case with $g = 1$ stands out because it also exhibits a pronounced intermediate stage in the correlation-learning regime where the relationship between Δ_θ and τ_θ does not follow the power-law tradeoff of the global bound with $\alpha \simeq 6$, but instead shows a stronger tradeoff with $\alpha \simeq \frac{1}{4}$. A more detailed analysis (see Sec. S5 D) suggests that this is caused by the emerging strong bimodal structure of the target distribution as g becomes smaller, which impedes and eventually prevents efficient learning.

D. Basis-dependent learning characteristics in the transverse-field Ising chain

We investigate the emergence of an intermediate stage in the correlation-learning regime, where learning is less efficient than admitted by the global power-law tradeoff, in the example of ground-state tomography for the transverse-field Ising chain in the σ^x basis as g becomes smaller (see, in particular, Fig. 2d of the main text).

Since there are some important differences in the learning characteristics of the TFIC ground-state distribution in the σ^z and σ^x bases (cf. Figs. 2b and d), we first examine the underlying target distribution $p(x)$ in more detail. To avoid confusion in the following, we denote the target distribution as $p^z(x)$ when referring to the σ^z basis representation and as $p^x(x)$ when referring to the σ^x basis representation. The Hamiltonian (S7) has a $\mathbb{Z}_2$ symmetry of the form $F := \prod_i \sigma_i^z$, i.e., $[H, F] = 0$. In the σ^z basis, F measures the parity of the number of down spins, whereas it describes a spin-flip symmetry in the σ^x basis.

For even M, to which we restrict our discussion exclusively in this work, the ground state (S12) of the Hamiltonian (S7) lies in the $F = +1$ sector. In the σ^z basis, this means that $p^z(x) = 0$ for half of the basis states, namely all configurations $x = (x_1, \ldots, x_M)$ for which $\sum_i x_i$ is odd. In the σ^x basis, by contrast, it implies that $p^x(x) = p^x(1 - x)$, where $1 - x = (1 - x_1, \ldots, 1 - x_M)$. This distinct manifestation of the symmetry F is the first important distinction between the σ^z- and σ^x-basis representations. Learning the σ^x representation is thus aided by the fact that the RBM model can encode *relative* spin orientations (alignment or anti-alignment) particularly efficiently, requiring only a single hidden unit for an arbitrary combination of visible ones, although the effectively needed number of weights appears to depend also on the proximity to the critical point [S9]. By contrast, due to the exponential form of the model distribution, it is rather inefficient at enforcing $\hat{p}_\theta(x) = 0$ for individual states x as required by the σ^z representation $p^z(x)$. These observations hint at possible origins of the different exponents α in the tradeoff relation (7) for the two representations, a large value $\alpha \simeq 6 \ldots 8$ for the better suited $p^x(x)$ and a small value $\alpha \simeq \frac{1}{2}$ for the worse suited $p^z(x)$.

For large values of g, $p^z(x)$ is dominated by the state $x = (0, \ldots, 0)$ ("all spins up"), while $p^x(x)$ becomes approximately uniform. For small values of g, in turn, $p^z(x)$ approaches a uniform distribution, whereas $p^x(x)$ is dominated by the two states $x = (0, \ldots, 0)$ and $x = (1, \ldots, 1)$, whose probability is degenerate due to the symmetry F. The different mode structure in the nonuniform limit of the σ^z- and σ^x-basis representations is a second important difference between them. Strongly unimodal as well as approximately uniform distributions are structurally simple, and gradient-descent training can find a globally optimal solution relatively easily. If the distribution has two (or a few) modes or dominant states, by contrast, the risk of getting stuck in a local minimum of the loss landscape and detecting only a subset of those dominant

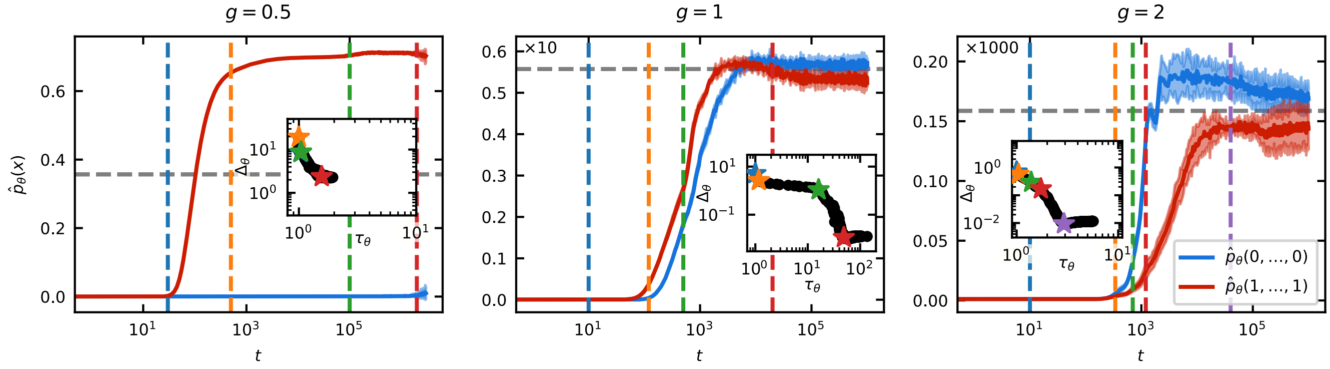

FIG. S9. Evolution of the model probabilities $\hat{p}_\theta(x)$ with the training time t for the two dominant states of the target distribution $p^x(x)$, $x = (0, \ldots, 0)$ (all spins up, blue) and $x = (1, \ldots, 1)$ (all spins down, red). Horizontal dashed lines show $p^x(x)$ for those states. Shaded areas indicate the fluctuations across five independent runs (standard deviation). Training used contrastive divergence of order $n_{\mathrm{CD}} = 1$ and hyperparameters as in Fig. 2d of the main paper, i.e., $\eta = 10^{-3}$, $B = 100$, $|S| = 25\,000$. Insets: Exact loss Δ_θ vs. autocorrelation time τ_θ. Selected epochs are marked by colored vertical dashed lines in the main panel and stars in the insets. Note the scaling of the y-axis in the middle and right panels as indicated in the top-left corner.

states is increased.

We presume that the bimodal structure of $p^x(x)$, which becomes increasingly pronounced as g becomes smaller, is the reason for the intermediate stage in the correlation-learning regime observed in the top panel ($g = 1$) of Fig. 2d. To substantiate this claim, we investigate the evolution of the model probability $\hat{p}_\theta(x)$ during training for the two modes $x^+ := (0, \ldots, 0)$ and $x^- := (1, \ldots, 1)$. Fig. S9 shows these probabilities for three different values of g.

We focus on the middle panel ($g = 1$) first, where $p^x(x^\pm) \approx 5.6\,\%$. The end of the correlation-learning regime is marked by the blue dashed line (blue star in the inset). Thereafter, the RBM first picks up the x^- mode, accompanied by a segment with $\alpha \simeq 6$ (see also Fig. 2d in the main text). The probability of the other mode x^+ only starts to increase slightly later at around the orange mark, which also corresponds to the starting point of the second stage of correlation learning with $\alpha \simeq \frac{1}{4}$. It lasts until both modes are established (green mark), and the subsequent fine tuning proceeds with a more efficient tradeoff exponent of $\alpha \simeq 6$ again. A similar, but less pronounced behavior can be observed for $g = 2$ (right panel of Fig. S9), where the two modes only have $p^x(x^\pm) \approx 0.015\,\%$. On the contrary, if the modes become more pronounced as exemplified by the $g = \frac{1}{2}$ case with $p^x(x^\pm) \approx 36\,\%$ (left panel), the RBM only learns one of the modes and fails to detect the other one.

SUPPLEMENTARY REFERENCES

[S1] G. E. Hinton, Training products of experts by minimizing contrastive divergence, Neural Comput. **14**, 1771 (2002).

[S2] G. E. Hinton, A practical guide to training restricted Boltzmann machines, in *Neural Networks: Tricks of the Trade: Second Edition*, edited by G. Montavon, G. B. Orr, and K.-R. Müller (Springer, Berlin, Heidelberg, 2012) pp. 599–619.

[S3] T. Tieleman, Training restricted Boltzmann machines using approximations to the likelihood gradient, in *Proceedings of the 25th International Conference on Machine Learning*, ICML '08 (Association for Computing Machinery, New York, NY, USA, 2008) p. 1064–1071.

[S4] A. Fischer and C. Igel, An introduction to restricted Boltzmann machines, in *Progress in Pattern Recognition, Image Analysis, Computer Vision, and Applications*, edited by L. Alvarez, M. Mejail, L. Gomez, and J. Jacobo (Springer, Berlin, Heidelberg, 2012) pp. 14–36.

[S5] G. Montúfar, Restricted Boltzmann machines: Introduction and review (2018), arXiv:1806.07066.

[S6] L. Vidmar and M. Rigol, Generalized Gibbs ensemble in integrable lattice models, J. Stat. Mech: Theory Exp. **2016**, 064007 (2016).

[S7] G. Torlai, G. Mazzola, J. Carrasquilla, M. Troyer, R. Melko, and G. Carleo, Neural-network quantum state tomography, Nat. Phys. **14**, 447 (2018).

[S8] Y. LeCun, C. Cortes, and C. J. C. Burges, The MNIST database of handwritten digits, `http://yann.lecun.com/exdb/mnist/`.

[S9] A. Golubeva and R. G. Melko, Pruning a restricted Boltzmann machine for quantum state reconstruction (2021), arXiv:2110.03676.
